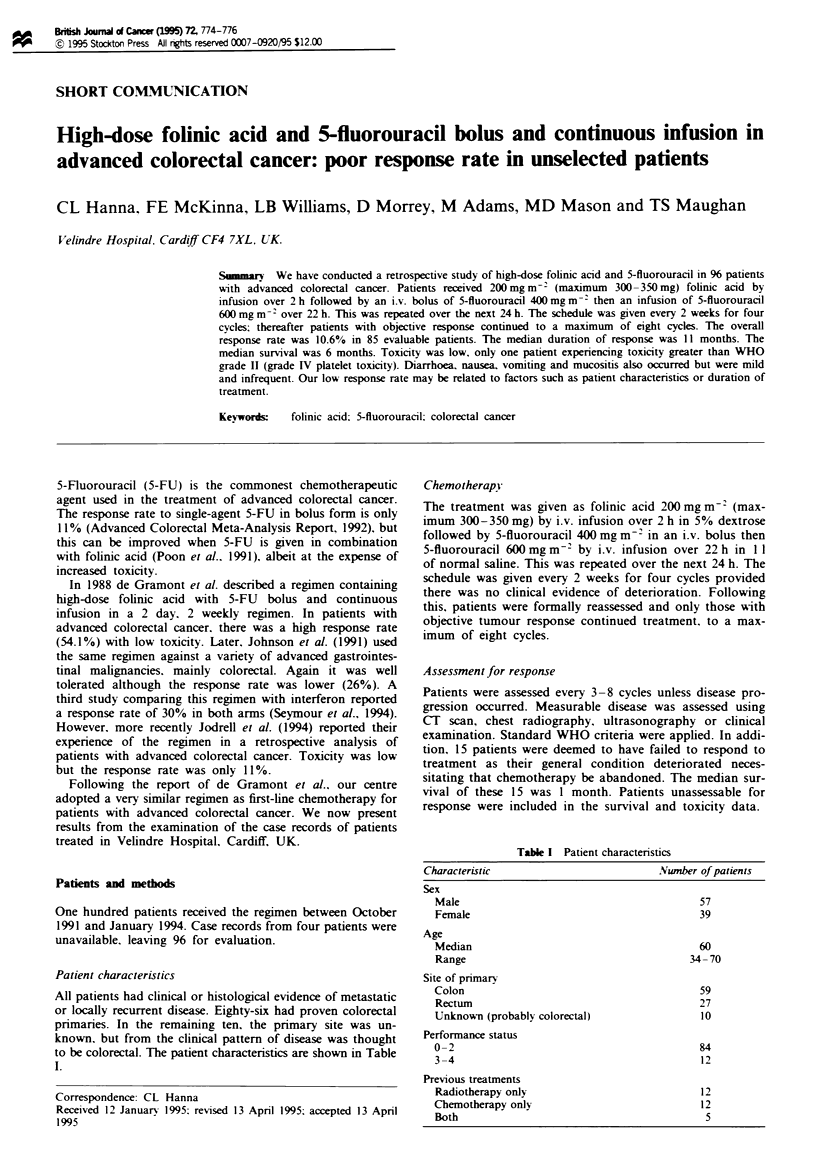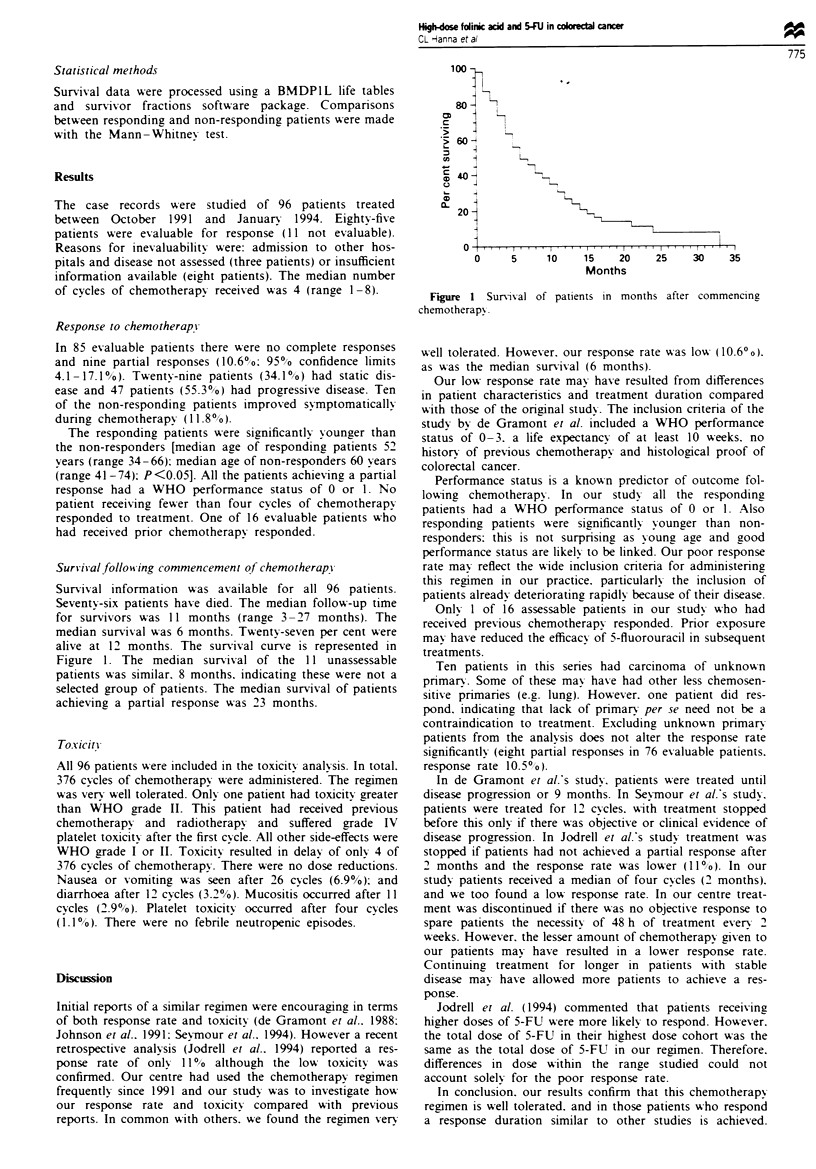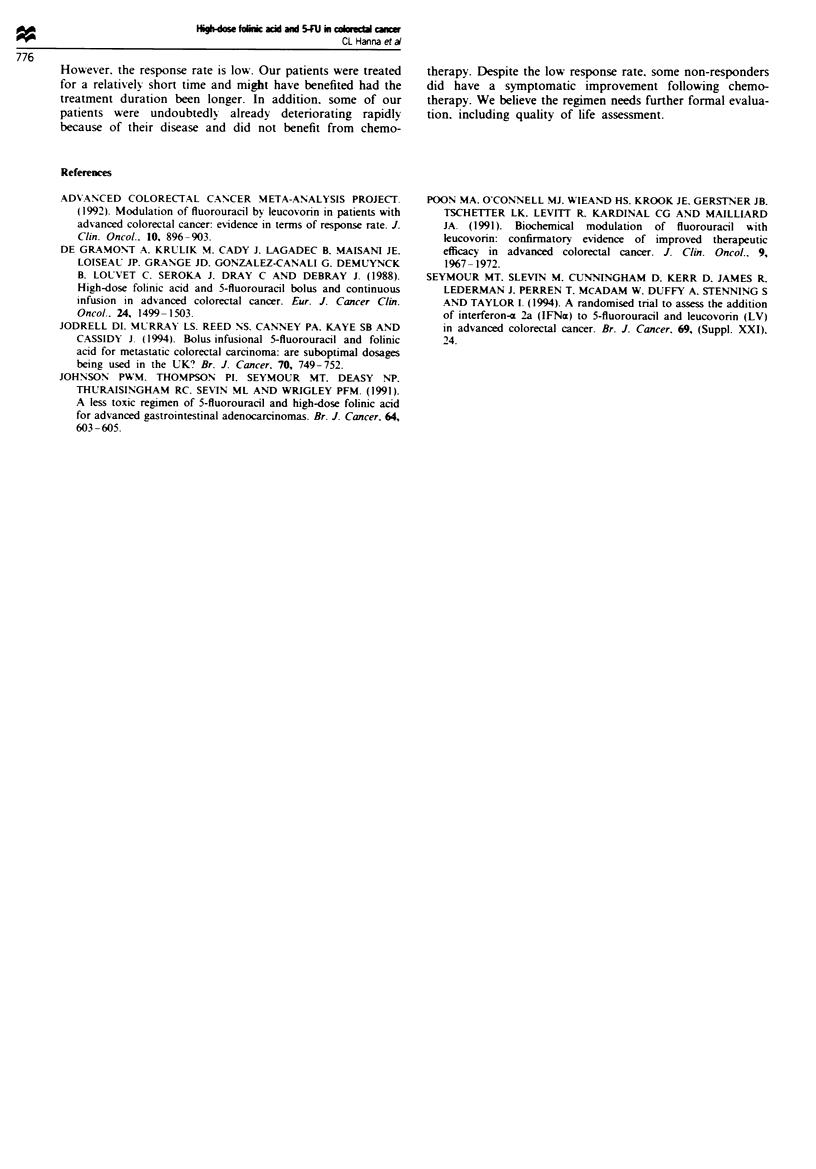# High-dose folinic acid and 5-fluorouracil bolus and continuous infusion in advanced colorectal cancer: poor response rate in unselected patients.

**DOI:** 10.1038/bjc.1995.409

**Published:** 1995-09

**Authors:** C. L. Hanna, F. E. McKinna, L. B. Williams, D. Morrey, M. Adams, M. D. Mason, T. S. Maughan

**Affiliations:** Velindre Hospital, Cardiff, UK.

## Abstract

We have conducted a retrospective study of high-dose folinic acid and 5-fluorouracil in 96 patients with advanced colorectal cancer. Patients received 200 mg m-2 (maximum 300-350 mg) folinic acid by infusion over 2 h followed by an i.v. bolus of 5-fluorouracil 400 mg m-2 then an infusion of 5-fluorouracil 600 mg m-2 over 22 h. This was repeated over the next 24 h. The schedule was given every 2 weeks for four cycles; thereafter patients with objective response continued to a maximum of eight cycles. The overall response rate was 10.6% in 85 evaluable patients. The median duration of response was 11 months. The median survival was 6 months. Toxicity was low, only one patient experiencing toxicity greater than WHO grade II (grade IV platelet toxicity). Diarrhoea, nausea, vomiting and mucositis also occurred but were mild and infrequent. Our low response rate may be related to factors such as patient characteristics or duration of treatment.


British Journal of Cancer (1995) 72, 774-776

© 1995 Stockton Press All rights reserved 0007-0920/95 $12.00

SHORT COMMUNICATION

### High-dose folinic acid and 5-fluorouracil bolus and continuous infusion in advanced colorectal cancer: poor response rate in unselected patients

CL Hanna, FE McKinna, LB Williams, D Morrey, M Adams, MD Mason and TS Maughan

*Velindre Hospital, Cardiff CF4 7XL, UK.*

**Summary** We have conducted a retrospective study of high-dose folinic acid and 5-fluorouracil in 96 patients with advanced colorectal cancer. Patients received 200 mg m$^{-2}$ (maximum 300-350 mg) folinic acid by infusion over 2 h followed by an i.v. bolus of 5-fluorouracil 400 mg m$^{-2}$ then an infusion of 5-fluorouracil 600 mg m$^{-2}$ over 22 h. This was repeated over the next 24 h. The schedule was given every 2 weeks for four cycles; thereafter patients with objective response continued to a maximum of eight cycles. The overall response rate was 10.6% in 85 evaluable patients. The median duration of response was 11 months. The median survival was 6 months. Toxicity was low, only one patient experiencing toxicity greater than WHO grade II (grade IV platelet toxicity). Diarrhoea, nausea, vomiting and mucositis also occurred but were mild and infrequent. Our low response rate may be related to factors such as patient characteristics or duration of treatment.

**Keywords:** folinic acid; 5-fluorouracil; colorectal cancer

5-Fluorouracil (5-FU) is the commonest chemotherapeutic agent used in the treatment of advanced colorectal cancer. The response rate to single-agent 5-FU in bolus form is only 11% (Advanced Colorectal Meta-Analysis Report, 1992), but this can be improved when 5-FU is given in combination with folinic acid (Poon *et al.*, 1991), albeit at the expense of increased toxicity.

In 1988 de Gramont *et al.* described a regimen containing high-dose folinic acid with 5-FU bolus and continuous infusion in a 2 day, 2 weekly regimen. In patients with advanced colorectal cancer, there was a high response rate (54.1%) with low toxicity. Later, Johnson *et al.* (1991) used the same regimen against a variety of advanced gastrointestinal malignancies, mainly colorectal. Again it was well tolerated although the response rate was lower (26%). A third study comparing this regimen with interferon reported a response rate of 30% in both arms (Seymour *et al.*, 1994). However, more recently Jodrell *et al.* (1994) reported their experience of the regimen in a retrospective analysis of patients with advanced colorectal cancer. Toxicity was low but the response rate was only 11%.

Following the report of de Gramont *et al.*, our centre adopted a very similar regimen as first-line chemotherapy for patients with advanced colorectal cancer. We now present results from the examination of the case records of patients treated in Velindre Hospital, Cardiff, UK.

### Patients and methods

One hundred patients received the regimen between October 1991 and January 1994. Case records from four patients were unavailable, leaving 96 for evaluation.

### *Patient characteristics*

All patients had clinical or histological evidence of metastatic or locally recurrent disease. Eighty-six had proven colorectal primaries. In the remaining ten, the primary site was unknown, but from the clinical pattern of disease was thought to be colorectal. The patient characteristics are shown in Table I.

Correspondence: CL Hanna
Received 12 January 1995; revised 13 April 1995; accepted 13 April 1995

### *Chemotherapy*

The treatment was given as folinic acid 200 mg m$^{-2}$ (maximum 300-350 mg) by i.v. infusion over 2 h in 5% dextrose followed by 5-fluorouracil 400 mg m$^{-2}$ in an i.v. bolus then 5-fluorouracil 600 mg m$^{-2}$ by i.v. infusion over 22 h in 1 l of normal saline. This was repeated over the next 24 h. The schedule was given every 2 weeks for four cycles provided there was no clinical evidence of deterioration. Following this, patients were formally reassessed and only those with objective tumour response continued treatment, to a maximum of eight cycles.

### *Assessment for response*

Patients were assessed every 3-8 cycles unless disease progression occurred. Measurable disease was assessed using CT scan, chest radiography, ultrasonography or clinical examination. Standard WHO criteria were applied. In addition, 15 patients were deemed to have failed to respond to treatment as their general condition deteriorated necessitating that chemotherapy be abandoned. The median survival of these 15 was 1 month. Patients unassessable for response were included in the survival and toxicity data.

**Table I** Patient characteristics

| *Characteristic* | *Number of patients* |
|---|---|
| Sex | |
| Male | 57 |
| Female | 39 |
| Age | |
| Median | 60 |
| Range | 34-70 |
| Site of primary | |
| Colon | 59 |
| Rectum | 27 |
| Unknown (probably colorectal) | 10 |
| Performance status | |
| 0-2 | 84 |
| 3-4 | 12 |
| Previous treatments | |
| Radiotherapy only | 12 |
| Chemotherapy only | 12 |
| Both | 5 |

### Statistical methods

Survival data were processed using a BMDP1L life tables and survivor fractions software package. Comparisons between responding and non-responding patients were made with the Mann–Whitney test.

### Results

The case records were studied of 96 patients treated between October 1991 and January 1994. Eighty-five patients were evaluable for response (11 not evaluable). Reasons for inevaluability were: admission to other hospitals and disease not assessed (three patients) or insufficient information available (eight patients). The median number of cycles of chemotherapy received was 4 (range 1–8).

### Response to chemotherapy

In 85 evaluable patients there were no complete responses and nine partial responses (10.6%; 95% confidence limits 4.1–17.1%). Twenty-nine patients (34.1%) had static disease and 47 patients (55.3%) had progressive disease. Ten of the non-responding patients improved symptomatically during chemotherapy (11.8%).

The responding patients were significantly younger than the non-responders [median age of responding patients 52 years (range 34–66); median age of non-responders 60 years (range 41–74); $P<0.05$]. All the patients achieving a partial response had a WHO performance status of 0 or 1. No patient receiving fewer than four cycles of chemotherapy responded to treatment. One of 16 evaluable patients who had received prior chemotherapy responded.

### Survival following commencement of chemotherapy

Survival information was available for all 96 patients. Seventy-six patients have died. The median follow-up time for survivors was 11 months (range 3–27 months). The median survival was 6 months. Twenty-seven per cent were alive at 12 months. The survival curve is represented in Figure 1. The median survival of the 11 unassessable patients was similar, 8 months, indicating these were not a selected group of patients. The median survival of patients achieving a partial response was 23 months.

### Toxicity

All 96 patients were included in the toxicity analysis. In total, 376 cycles of chemotherapy were administered. The regimen was very well tolerated. Only one patient had toxicity greater than WHO grade II. This patient had received previous chemotherapy and radiotherapy and suffered grade IV platelet toxicity after the first cycle. All other side-effects were WHO grade I or II. Toxicity resulted in delay of only 4 of 376 cycles of chemotherapy. There were no dose reductions. Nausea or vomiting was seen after 26 cycles (6.9%); and diarrhoea after 12 cycles (3.2%). Mucositis occurred after 11 cycles (2.9%). Platelet toxicity occurred after four cycles (1.1%). There were no febrile neutropenic episodes.

**Figure 1** Survival of patients in months after commencing chemotherapy.

### Discussion

Initial reports of a similar regimen were encouraging in terms of both response rate and toxicity (de Gramont *et al.*, 1988; Johnson *et al.*, 1991; Seymour *et al.*, 1994). However a recent retrospective analysis (Jodrell *et al.*, 1994) reported a response rate of only 11% although the low toxicity was confirmed. Our centre had used the chemotherapy regimen frequently since 1991 and our study was to investigate how our response rate and toxicity compared with previous reports. In common with others, we found the regimen very well tolerated. However, our response rate was low (10.6%), as was the median survival (6 months).

Our low response rate may have resulted from differences in patient characteristics and treatment duration compared with those of the original study. The inclusion criteria of the study by de Gramont *et al.* included a WHO performance status of 0–3, a life expectancy of at least 10 weeks, no history of previous chemotherapy and histological proof of colorectal cancer.

Performance status is a known predictor of outcome following chemotherapy. In our study all the responding patients had a WHO performance status of 0 or 1. Also responding patients were significantly younger than non-responders; this is not surprising as young age and good performance status are likely to be linked. Our poor response rate may reflect the wide inclusion criteria for administering this regimen in our practice, particularly the inclusion of patients already deteriorating rapidly because of their disease.

Only 1 of 16 assessable patients in our study who had received previous chemotherapy responded. Prior exposure may have reduced the efficacy of 5-fluorouracil in subsequent treatments.

Ten patients in this series had carcinoma of unknown primary. Some of these may have had other less chemosensitive primaries (e.g. lung). However, one patient did respond, indicating that lack of primary *per se* need not be a contraindication to treatment. Excluding unknown primary patients from the analysis does not alter the response rate significantly (eight partial responses in 76 evaluable patients, response rate 10.5%).

In de Gramont *et al.*'s study, patients were treated until disease progression or 9 months. In Seymour *et al.*'s study, patients were treated for 12 cycles, with treatment stopped before this only if there was objective or clinical evidence of disease progression. In Jodrell *et al.*'s study treatment was stopped if patients had not achieved a partial response after 2 months and the response rate was lower (11%). In our study patients received a median of four cycles (2 months), and we too found a low response rate. In our centre treatment was discontinued if there was no objective response to spare patients the necessity of 48 h of treatment every 2 weeks. However, the lesser amount of chemotherapy given to our patients may have resulted in a lower response rate. Continuing treatment for longer in patients with stable disease may have allowed more patients to achieve a response.

Jodrell *et al.* (1994) commented that patients receiving higher doses of 5-FU were more likely to respond. However, the total dose of 5-FU in their highest dose cohort was the same as the total dose of 5-FU in our regimen. Therefore, differences in dose within the range studied could not account solely for the poor response rate.

In conclusion, our results confirm that this chemotherapy regimen is well tolerated, and in those patients who respond a response duration similar to other studies is achieved.

However, the response rate is low. Our patients were treated for a relatively short time and might have benefited had the treatment duration been longer. In addition, some of our patients were undoubtedly already deteriorating rapidly because of their disease and did not benefit from chemotherapy. Despite the low response rate, some non-responders did have a symptomatic improvement following chemotherapy. We believe the regimen needs further formal evaluation, including quality of life assessment.

### References

ADVANCED COLORECTAL CANCER META-ANALYSIS PROJECT. (1992). Modulation of fluorouracil by leucovorin in patients with advanced colorectal cancer: evidence in terms of response rate. *J. Clin. Oncol.*, **10**, 896-903.

DE GRAMONT A, KRULIK M, CADY J, LAGADEC B, MAISANI JE, LOISEAU JP, GRANGE JD, GONZALEZ-CANALI G, DEMUYNCK B, LOUVET C, SEROKA J, DRAY C AND DEBRAY J. (1988). High-dose folinic acid and 5-fluorouracil bolus and continuous infusion in advanced colorectal cancer. *Eur. J. Cancer Clin. Oncol.*, **24**, 1499-1503.

JODRELL DI, MURRAY LS, REED NS, CANNEY PA, KAYE SB AND CASSIDY J. (1994). Bolus infusional 5-fluorouracil and folinic acid for metastatic colorectal carcinoma: are suboptimal dosages being used in the UK? *Br. J. Cancer*, **70**, 749-752.

JOHNSON PWM, THOMPSON PI, SEYMOUR MT, DEASY NP, THURAISINGHAM RC, SEVIN ML AND WRIGLEY PFM. (1991). A less toxic regimen of 5-fluorouracil and high-dose folinic acid for advanced gastrointestinal adenocarcinomas. *Br. J. Cancer*, **64**, 603-605.

POON MA, O'CONNELL MJ, WIEAND HS, KROOK JE, GERSTNER JB, TSCHETTER LK, LEVITT R, KARDINAL CG AND MAILLIARD JA. (1991). Biochemical modulation of fluorouracil with leucovorin: confirmatory evidence of improved therapeutic efficacy in advanced colorectal cancer. *J. Clin. Oncol.*, **9**, 1967-1972.

SEYMOUR MT, SLEVIN M, CUNNINGHAM D, KERR D, JAMES R, LEDERMAN J, PERREN T, MCADAM W, DUFFY A, STENNING S AND TAYLOR I. (1994). A randomised trial to assess the addition of interferon-α 2a (IFNα) to 5-fluorouracil and leucovorin (LV) in advanced colorectal cancer. *Br. J. Cancer*, **69**, (Suppl. XXI), 24.